# 

**Published:** 1895-08

**Authors:** 


### TABLE OF CONTENTS.

ORIGINAL COMMUNICATIONS. PAGE

Dental and Facial Orthopædia. By CALVIN S. CASE, D.D.S., M.D., Chicago . . . . . 463

Salol. By R. M. SANGER, D.D.S., Orange, N. J. . . . . 472

Impacted Third Molars. By DR. J. W. FOREMAN, Asheville, N. C. . . . 475

The Rational Therapeutics of Pyorrhœa Alveolaris. By HENRY H. BURCHARD, M.D., D.D.S., Philadelphia . . . . . 477

Dentistry in the Public Schools. By E. PROCTOR HOLMES, D.M.D., Stoughton, Mass. . . . . . 482

A Consideration of Some Problems in a Dentist's Life. By EZRA F. TAFT, D.M.D., Cambridge, Mass. . . . . . 486

REPORTS OF SOCIETY MEETINGS.

New York Odontological Society . . . . . 491

Odontological Society of Pennsylvania . . . . . 514

EDITORIAL.

Will Dentistry be absorbed by Medicine? . . . . . 517

BIBLIOGRAPHY . . . . . 519

OBITUARY.

Resolutions on the Death of Dr. J. J. R. Patrick . . . . . 522

NOTES AND COMMENTS . . . . . 522

CURRENT NEWS.

American Dental Association . . . . . 523

Harvard Dental Alumni Association . . . . . 524

Woman's Dental Association . . . . . 525

Illinois State Dental Society . . . . . 525

Alumni Association, University of Michigan . . . . . 526

Dental Society of the State of New York . . . . . 526

